# Phage Resistance Accompanies Reduced Fitness of Uropathogenic Escherichia coli in the Urinary Environment

**DOI:** 10.1128/msphere.00345-22

**Published:** 2022-08-03

**Authors:** Jacob J. Zulk, Justin R. Clark, Samantha Ottinger, Mallory B. Ballard, Marlyd E. Mejia, Vicki Mercado-Evans, Emmaline R. Heckmann, Belkys C. Sanchez, Barbara W. Trautner, Anthony W. Maresso, Kathryn A. Patras

**Affiliations:** a Department of Molecular Virology and Microbiology, Baylor College of Medicine, Houston, Texas, USA; b Medical Scientist Training Program, Baylor College of Medicine, Houston, Texas, USA; c Center for Innovations in Quality, Effectiveness, and Safety (IQuESt), Michael E. DeBakey Veterans Affairs Medical Center, Houston, Texas, USA; d Section of Health Services Research, Department of Medicine, Baylor College of Medicine, Houston, Texas, USA; e Alkek Center for Metagenomics and Microbiome Research, Baylor College of Medicine, Houston, Texas, USA; University of Kentucky

**Keywords:** antimicrobial resistance, bacteriophage therapy, urinary tract infection, uropathogenic *E. coli*

## Abstract

Urinary tract infection (UTI) is among the most common infections treated worldwide each year and is caused primarily by uropathogenic Escherichia coli (UPEC). Rising rates of antibiotic resistance among uropathogens have spurred a consideration of alternative treatment strategies, such as bacteriophage (phage) therapy; however, phage-bacterial interactions within the urinary environment are poorly defined. Here, we assess the activity of two phages, namely, HP3 and ES17, against clinical UPEC isolates using *in vitro* and *in vivo* models of UTI. In both bacteriologic medium and pooled human urine, we identified phage resistance arising within the first 6 to 8 h of coincubation. Whole-genome sequencing revealed that UPEC strains resistant to HP3 and ES17 harbored mutations in genes involved in lipopolysaccharide (LPS) biosynthesis. Phage-resistant strains displayed several *in vitro* phenotypes, including alterations to adherence to and invasion of human bladder epithelial HTB-9 cells and increased biofilm formation in some isolates. Interestingly, these phage-resistant UPEC isolates demonstrated reduced growth in pooled human urine, which could be partially rescued by nutrient supplementation and were more sensitive to several outer membrane-targeting antibiotics than parental strains. Additionally, phage-resistant UPEC isolates were attenuated in bladder colonization in a murine UTI model. In total, our findings suggest that while resistance to phages, such as HP3 and ES17, may arise readily in the urinary environment, phage resistance is accompanied by fitness costs which may render UPEC more susceptible to host immunity or antibiotics.

**IMPORTANCE** UTI is one of the most common causes of outpatient antibiotic use, and rising antibiotic resistance threatens the ability to control UTI unless alternative treatments are developed. Bacteriophage (phage) therapy is gaining renewed interest; however, much like with antibiotics, bacteria can readily become resistant to phages. For successful UTI treatment, we must predict how bacteria will evade killing by phage and identify the downstream consequences of phage resistance during bacterial infection. In our current study, we found that while phage-resistant bacteria quickly emerged *in vitro*, these bacteria were less capable of growing in human urine and colonizing the murine bladder. These results suggest that phage therapy poses a viable UTI treatment if phage resistance confers fitness costs for the uropathogen. These results have implications for developing cocktails of phage with multiple different bacterial targets, of which each is evaded only at the cost of bacterial fitness.

## INTRODUCTION

Urinary tract infection (UTI) is an extremely common bacterial infection, causing nearly 10 million cases in the United States alone each year (1, 2). These infections disproportionately affect women, with approximately half of women experiencing at least one UTI during their lifetime (3). Uropathogenic Escherichia coli (UPEC) is the leading cause of UTI worldwide, causing upward of 75% of infections. UTI is one of the most common causes of outpatient antibiotic prescriptions (4, 5), and the rise of antibiotic resistance among UPEC isolates threatens existing treatments for UTI (6, 7). Current technological and economic challenges limit the development of novel antibiotics, and antibiotic resistance develops rapidly once antibiotics are introduced (8–10). Because of these challenges, several new nonantibiotic alternatives to treat UPEC UTI have been proposed (11–17). One such alternative are bacteriophages (phages), which are viruses that use bacteria as their natural host.

Soon after the discovery of phages in the early 1900s, phage therapy was applied to bacterial infections (18), including UTI caused by UPEC (19). Despite this early interest, phage therapy was abandoned largely in favor of antibiotics, but with the modern rise in antibiotic-resistant infections, interest in phage therapy is resurging (20, 21). Phage therapy holds several appealing properties. Bacteriophages outnumber bacteria by an estimated 10:1 ratio (22), and phages that target human pathogens are isolated readily from environmental and human sources (23–25). Additionally, phages replicate within the bacterial host, generating a source of new phages for the duration of the pathogen presence (self-dosing). Moreover, phages may have fewer off-target impacts on the host microbiota (26). To date, phage therapy for UTI has been confined to compassionate care use (27–31) and has shown generally favorable results. Clinical trials testing dosing and administration methods for UTI phage therapy are currently in the early stages of development (32, 33). A randomized clinical trial conducted on UPEC UTI described phage therapy as noninferior to standard-of-care antibiotics but also nonsuperior to placebo treatment (34). Factors specific to the urinary environment or to uropathogens, such as UPEC, may impact phage efficacy, and thus, understanding phage-bacterial interactions in the urinary tract is critical to drive UTI phage therapies.

A key challenge to phage therapy efficacy is the emergence of bacterial resistance toward phage killing. Similar to evolving resistance to antibiotics, bacteria can rapidly develop resistance to phage. Resistance mechanisms include blocking of phage adsorption through the mutation of phage receptors, masking of phage targets, and producing competitive inhibitors (35, 36). Additionally, even after phage internalization, bacteria can resist phage infection by blocking DNA entry or degrading viral DNA intracellularly (37). Phage resistance is also associated with reduced bacterial fitness, particularly related to phage receptor modification (35, 38). Fitness costs include reduced virulence and increased susceptibility to antimicrobials (35, 39–43) or immune clearance (44). Phage resistance paired with reduced virulence suggests that “steering” bacteria toward these phenotypes may be a viable approach for treating infections (44). Despite the current knowledge on phage resistance and potential fitness costs, the effect of phage resistance in uropathogens within the urinary tract has not been assessed.

In this study, we evaluate mechanisms of UPEC phage resistance to two genetically distinct bacteriophages (24) and test the hypothesis that phage resistance may bring associated bacterial fitness costs within the urinary environment. Through unbiased screening and sequencing, we found that UPEC resistance to phages HP3 and ES17 is associated with mutations in the lipopolysaccharide (LPS) biosynthesis pathway. Phage resistance mutations negatively impact UPEC growth in urine and resistance to membrane-targeting antibiotics and result in reduced colonization of the urinary tract *in vivo*. Together, these findings provide insight into mechanisms and associated impacts of phage resistance during uropathogenesis and suggest phage-associated mutations reduce UPEC fitness in the urinary tract. These findings provide a critical knowledge base for the future development of UTI phage therapy.

## RESULTS

### UPEC strains resistant to phage emerge rapidly *in vitro* in LB and human urine.

Conventionally, bacterium-phage interactions are studied in bacteriologic medium; however, recent work has shifted to assess these interactions in the context of the host, including *ex vivo* blood or *in vivo* tissues (44–46). To assess how phage activity is influenced by the urinary environment, we compared the susceptibility of four UPEC strains to two distinct phages, namely, HP3 and ES17 (24, 31) either in LB medium or human urine. These phage target extraintestinal pathogenic E. coli (ExPEC), including UPEC (24, 31, 35, 47, 48). For UPEC strains, we selected well-characterized UPEC cystitis and pyelonephritis isolates (UTI89 and CFT073, respectively) and DS515 and DS566, which are recent isolates from patients with neurogenic bladders from spinal cord injury.

**FIG 1:**
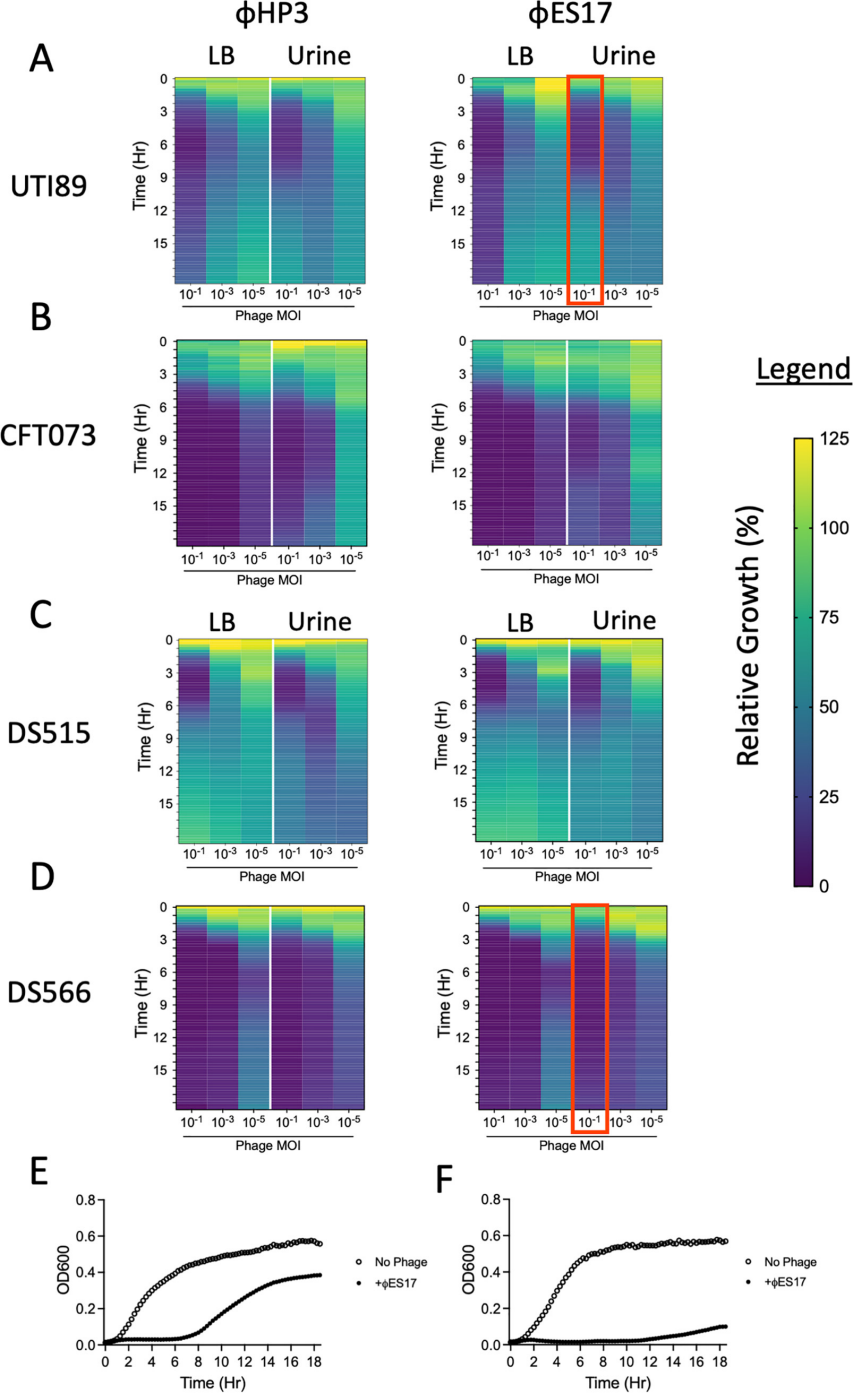
Phage-bacterial dynamics are similar in LB and pooled human urine with resistance developing under both conditions. Heatmaps of relative bacterial growth (OD_600_ of phage treated well/OD_600_ of untreated well) of bacteria challenged with HP3 (left column) or ES17 (right column) in LB media and pooled human urine during 18 h of growth. Data are grouped by UPEC strain UTI89 (A), CFT073 (B), DS515 (C), and DS566 (D). All bacteria were challenged at multiplicities of infection (MOIs) of 10^−1^, 10^−3^, and 10^−5^. (E) Representative growth curve of UTI89 challenged with ES17 at a MOI of 10^−1^ in urine highlighted with red box in A. (F) Representative growth curve of DS566 challenged with ES17 at MOI of 10^−1^ in urine highlighted with red box in D. All heatmaps are representative of three independent experiments performed in at least technical duplicates. Growth curves for these data appear in Fig. S1 and S3.

Approximately 10^7^ CFU of UPEC were challenged with 10^6^, 10^4^, or 10^2^ PFU of phage (multiplicity of infection [MOI] of 10^−1^, 10^−3^, and 10^−5^, respectively) in 96-well microtiter plates. Within 1 to 2 h of phage challenge, the relative growth of phage-treated wells declined rapidly. Minimal growth (<25% growth of optical density at 600 nm [OD_600_] in treated versus untreated wells) was observed for the first ~6 h in the presence of the two higher MOIs for both phages (Fig. 1A to D; see Fig. S1 to S3 in the supplemental material). After this time, many of the cultures began to “rebound” their relative growth, suggesting bacterial resistance toward the phage. This activity was observed most strongly in strains UTI89 (Fig. 1A) and DS515 (Fig. 1C). There were no clear differences in relative growth based on the medium used (LB versus urine), although a decreasing phage MOI reduced the relative growth suppression. UTI89 and DS566 were selected for further investigation into the mechanisms of phage resistance based on the differential growth kinetics observed during phage challenge. While relative growth rebounded quickly in phage-challenged strains (Fig. 1E), DS566 relative growth was slower, reaching only ~20% of no-phage control growth after 18 h (Fig. 1F).

10.1128/msphere.00345-22.1 FIG S1 Growth of UPEC in the presence of phages HP3 and ES17 at an MOI of 10^−1^. Bacteria were challenged with HP3 (left column) or ES17 (right column) in LB media and pooled human urine during 18 h of growth. Data are grouped by UPEC strain UTI89 (A), CFT073 (B), DS515 (C), and DS566 (D). All curves represent the mean value of three independent experiments performed in at least technical duplicate. Download FIG S1, JPG file, 0.3 MB. Copyright © 2022 Zulk et al. 2022 Zulk et al. https://creativecommons.org/licenses/by/4.0/ This content is distributed under the terms of the Creative Commons Attribution 4.0 International license.

10.1128/msphere.00345-22.2 FIG S2 Growth of UPEC in the presence of phages HP3 and ES17 at an MOI of 10^−3^. Bacteria were challenged with HP3 (left column) or ES17 (right column) in LB media and pooled human urine during 18 h of growth. Data are grouped by UPEC strain UTI89 (A), CFT073 (B), DS515 (C), and DS566 (D). All curves represent the mean value of three independent experiments performed in at least technical duplicate. Download FIG S2, JPG file, 0.3 MB. Copyright © 2022 Zulk et al. 2022 Zulk et al. https://creativecommons.org/licenses/by/4.0/ This content is distributed under the terms of the Creative Commons Attribution 4.0 International license.

10.1128/msphere.00345-22.3 FIG S3 Growth of UPEC in the presence of phages HP3 and ES17 at an MOI of 10^−5^. Bacteria were challenged with HP3 (left column) or ES17 (right column) in LB media and pooled human urine during 18 h of growth. Data are grouped by UPEC strain UTI89 (A), CFT073 (B), DS515 (C), and DS566 (D). All curves represent the mean value of three independent experiments performed in at least technical duplicate. Download FIG S3, JPG file, 0.3 MB. Copyright © 2022 Zulk et al. 2022 Zulk et al. https://creativecommons.org/licenses/by/4.0/ This content is distributed under the terms of the Creative Commons Attribution 4.0 International license.

### Phage resistance is associated with mutations in lipopolysaccharide (LPS) biosynthesis.

**FIG 2:**
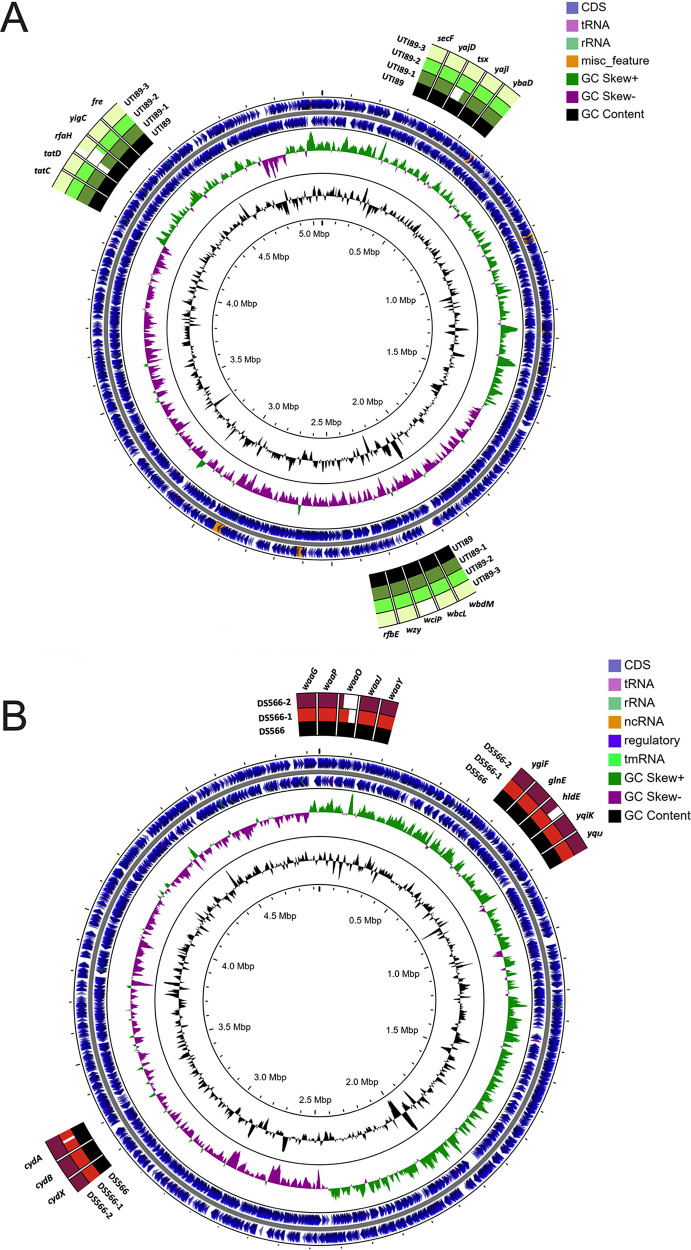
Whole-genome sequencing of phage-resistant bacteria identifies multiple mutations in UPEC strains UTI89 and DS566 exposed to phages HP3 and ES17. Alignment of whole-genome sequences depicting mutations identified in UTI89 phage-resistant mutants (A) and DS566 phage-resistant mutants (B) compared with that of parental wild-type (WT) strains.

**FIG 3:**
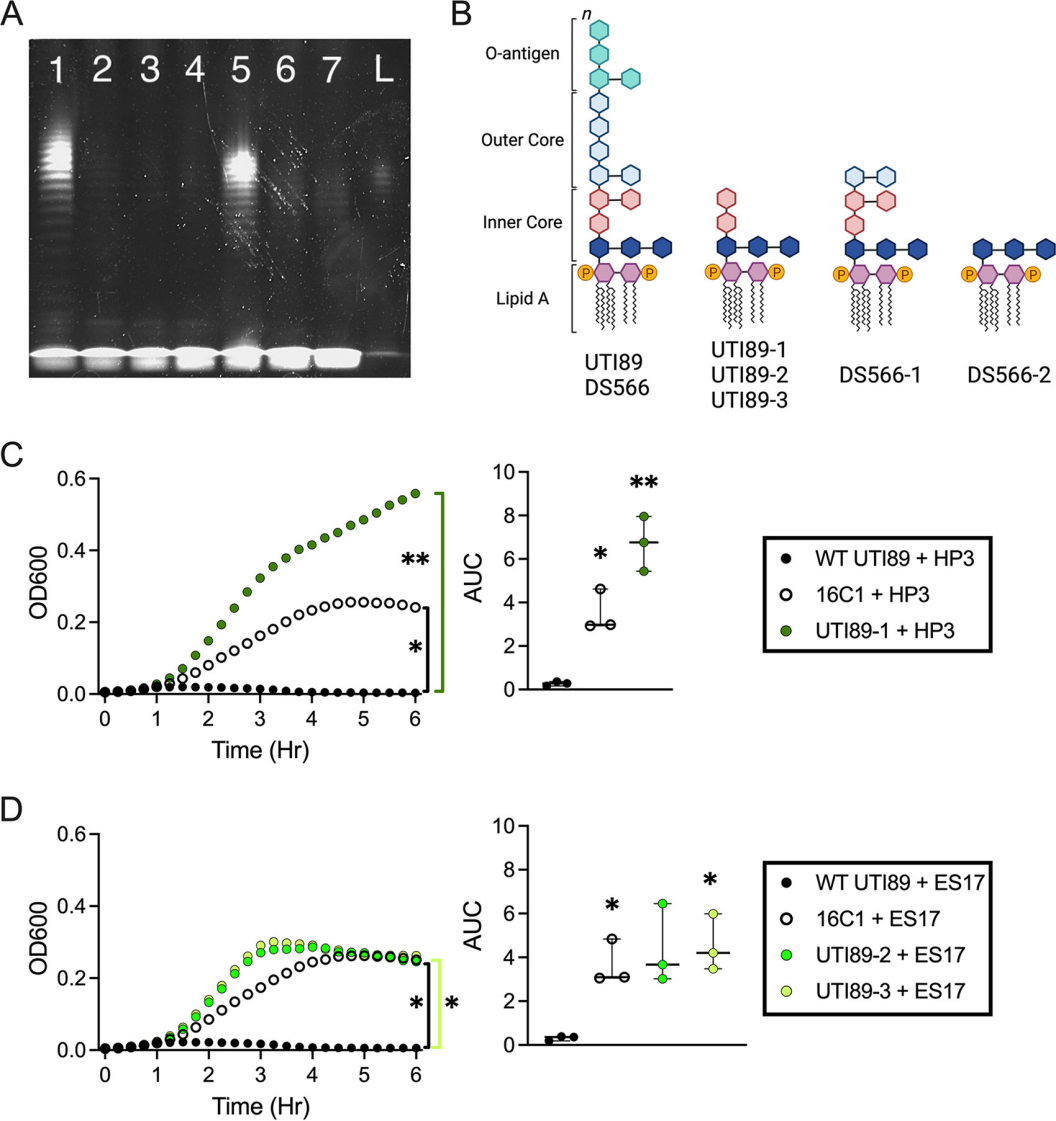
Phage resistance to HP3 and ES17 is associated with LPS deficiency. (A) Representative SDS-PAGE image of WT and phage-resistant UPEC LPS isolated through hot aqueous-phenol extraction and run on a 4% to 12% SDS-polyacrylamide gel loaded with 15 μL of isolated product. Lane 1, WT UTI89; lane 2, UTI89-1; lane 3, UTI89-2; lane 4, UTI89-3; lane 5, WT DS566; lane 6, DS566-1; lane 7, DS566-2; L, LPS standard. Isolation and visualization of LPS was performed in two independent experiments with comparable results. (B) Predicted LPS structures based on gene mutations noted in the sequencing analysis. (C and D) Growth of WT UTI89, *rfa* transposon mutant 16C1, or phage-resistant UTI89 in the presence of HP3 (C) or ES17 (D) over 6 h. Growth curves of bacteria in the presence of phage are representative of the mean value of three independent experiments, with each performed with four technical replicates. Bars represent median and 95% confidence intervals (CIs). Growth curves were analyzed by repeated measures two-way ANOVA with Geisser-Greenhouse correction and Dunnett’s multiple-comparison test. *, *P < *0.05; **, *P < *0.01.

To isolate phage-resistant UPEC, bacteria were passaged a total of four times in the presence of phage in liquid culture and on agar containing phage. Three UTI89 isolates resistant to HP3 or ES17 and two DS566 isolates resistant to ES17 were subjected to whole-genome sequencing. All UTI89 isolates resistant to phage, regardless of which phage, had mutations in the transcription factor rfaH (locus tag b3842) (Fig. 2A, Table 1). DS566 strains had mutations in galactotransferase rfaI/waaO (locus tag b3627). DS566-2 had an additional mutation in rfaE/hldE (locus tag b3052) (Fig. 2B; Table 1). Several non-LPS-related mutations were also observed (see Table S1 in the supplemental material). To visualize changes to LPS in phage-resistant strains, LPS was isolated via hot aqueous-phenol extraction and subjected to SDS-PAGE. Loss of the LPS O-antigen was apparent in all phage-resistant strains compared with the parental strains (Fig. 3A). These results agree with predicted LPS structures in rfaH and rfaE/hldE mutants (truncated inner core), while rfaI/waaO is likely to result in outer core truncation (49) (Fig. 3B). Phage-resistant strain UTI89-2 was serially passaged three times, and no sensitivity to phage ES17 was regained (see Fig. S4 in the supplemental material), suggesting that these mutations are stable even in the absence of selective pressure. To test whether a specific mutation of LPS biosynthesis could confer resistance to phages HP3 or ES17, we challenged UTI89 16C1, which harbors a transposon insertion in the *rfa* operon, with phage. Strain 16C1, as well as phage-resistant strain UTI89-1, grew significantly better than wild-type UTI89 in the presence of HP3 (*P *= 0.023 and *P *= 0.0046, respectively) (Fig. 3C). Interestingly, UTI89-1 growth was enhanced compared with 16C1 when challenged with HP3, suggesting a mutation of the promoter *rfaH* may confer more resistance than a mutation to the *rfa* operon itself. In the presence of ES17, 16C1 also grew significantly better than wild-type UTI89 (*P *= 0.041) (Fig. 3D). Similarly, phage-resistant strains UTI89-2 and UTI89-3 achieved greater growth than wild-type UTI89 (*P *= 0.092 and *P *= 0.029, respectively) (Fig. 3D).

**TABLE 1:** LPS biosynthesis-associated mutations observed in phage-resistant UPEC^*a*^

Strain	Phage resistant against	Gene(s) mutated	Earliest LPS mutation	Predicted effect
UTI89-1	HP3	*rfaH*	rfaH	Truncated LPS inner core
UTI89-2	ES17	*rfaH*	rfaH	Truncated LPS inner core
UTI89-3	ES17	*rfaH*, *wbbL*	rfaH	Truncated LPS inner core
DS566-1	ES17	*rfaI*/*waaO*	rfaI/waaO	Truncated LPS outer core
DS566-2	ES17	*rfaI*/*waaO*, *rfaE*/*hldE*	rfaE/hldE	Truncated LPS inner core

a Mutations associated with LPS biosynthesis in phage-resistant UPEC were identified through whole-genome sequencing. The site and type of LPS biosynthesis-associated mutations were observed in phage-resistant strains arising from *in vitro* screening and were identified by whole-genome sequencing.

10.1128/msphere.00345-22.4 FIG S4 Serially passaged phage-resistant UPEC strains do not readily regain sensitivity to ES17. Three independent cultures of phage-resistant strain UTI89-2 were serially passaged three times in LB media. Each passage, the parental UTI89-2 strain and wild-type UTI89 were challenged with ES17 for 6 h. Data are grouped by passage number 0 (A), 1 (B), 2 (C), and 3 (D). Each growth curve represents the mean value of three technical replicates. Download FIG S4, JPG file, 0.3 MB. Copyright © 2022 Zulk et al. 2022 Zulk et al. https://creativecommons.org/licenses/by/4.0/ This content is distributed under the terms of the Creative Commons Attribution 4.0 International license.

10.1128/msphere.00345-22.6 TABLE S1 Non-LPS-associated mutations identified during sequencing of phage-resistant isolates. Site and type of non-LPS biosynthesis-associated mutations observed in phage-resistant strains arising from *in vitro* screening and identified by whole-genome sequencing. Download Table S1, DOCX file, 0.1 MB. Copyright © 2022 Zulk et al. 2022 Zulk et al. https://creativecommons.org/licenses/by/4.0/ This content is distributed under the terms of the Creative Commons Attribution 4.0 International license.

### Phage resistance is associated with altered bacterial biofilm formation and adherence and invasion of bladder cells.

**FIG 4:**
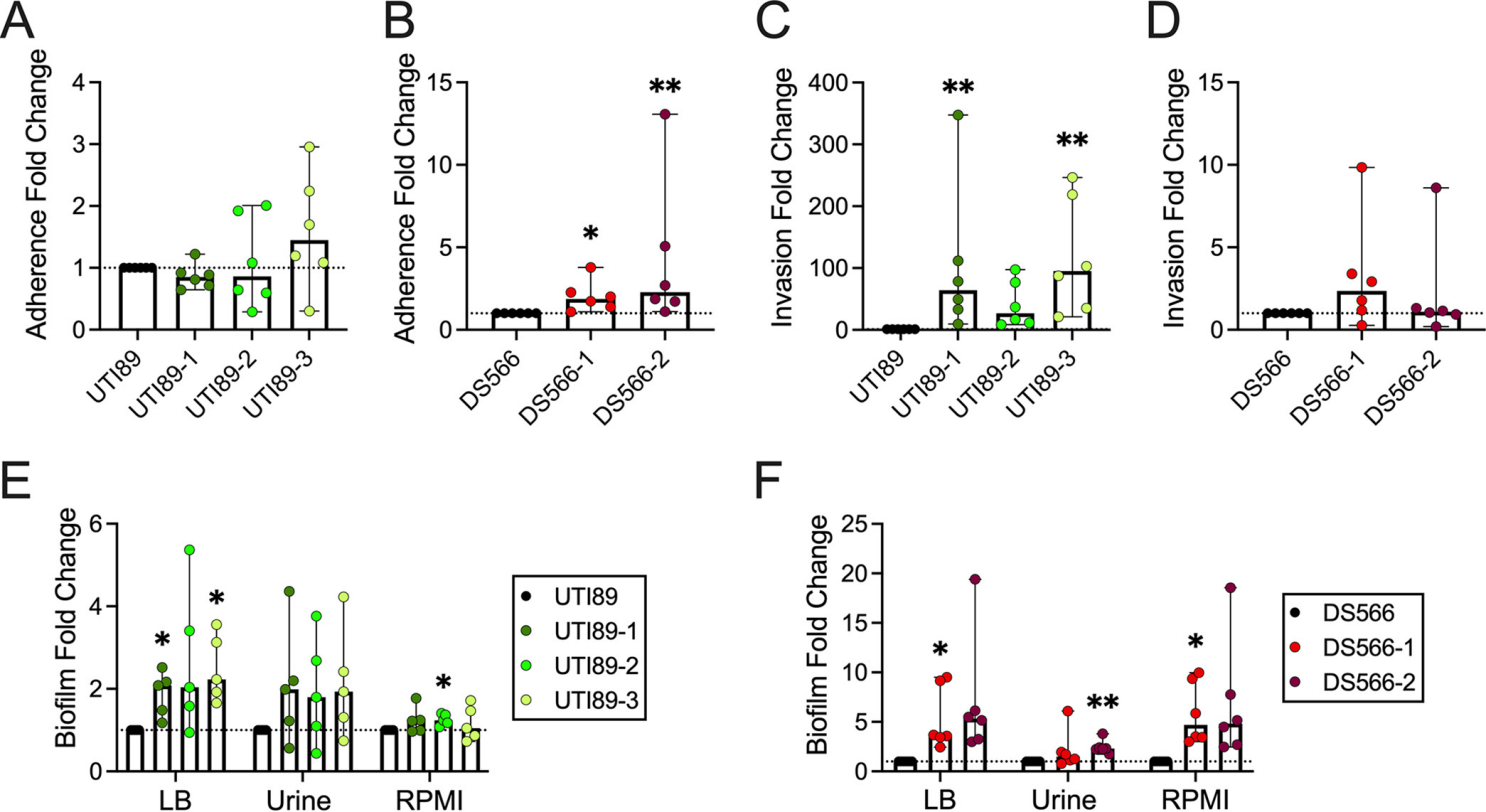
Adherence and invasion of HTB-9 cells as well as biofilm formation are altered in phage-resistant UPEC. (A and B) UTI89 (A) and DS566 (B) and respective phage-resistant strain adherence to HTB-9 cells after 30 min of infection; MOI of 1. HTB-9 cells were infected with UTI89 (C) or DS566 (D) and respective phage-resistant mutants (MOI of 1) for 2 h before the medium was changed to medium containing antibiotics to kill extracellular bacteria. After 2 h of antibiotic treatment, cells were lysed and intracellular bacteria enumerated. Biofilm formation of UTI89 (E) or DS566 (F) and respective phage-resistant mutants in LB, urine, or RPMI 1640 quantified by crystal violet uptake. All adherence, invasion, and biofilm assays were performed in at least technical duplicate in five to six independent experiments, with points on graphs representing mean values of each independent experiment. Bars represent median and 95% confidence intervals. Data were analyzed by Kruskal-Wallis test using Dunn’s multiple-comparison test (A to D) or repeated-measures two-way ANOVA with Geisser-Greenhouse correction and Dunnett’s multiple-comparison test (E, F). *, *P < *0.05; **, *P < *0.01.

UPEC adheres to and invades the bladder epithelium to form intracellular bacterial reservoirs capable of reseeding infection (50, 51); thus, we assessed if phage resistance alters UPEC interactions with HTB-9 bladder cells. No change in adherence was seen in any of the UTI89 phage-resistant strains (Fig. 4A); however, both DS566 strains had modestly increased adherence relative to the wild type, with a 1.9-fold increase for DS566-1 (*P* = 0.014) and 2.3-fold increase for DS566-2 (*P* = 0.0023) (Fig. 4B). Invasion of HTB-9 cells was increased 50- to 100-fold in 2 UTI89 strains, namely, UTI89-1 and UTI89-3 (*P *= 0.0053 and *P *= 0.0012, respectively) (Fig. 4C), while no change in invasion was detected in the DS566 strains (Fig. 4D).

As LPS modifications may change bacterial surface properties (52, 53), we evaluated the ability of phage-resistant strains to form biofilms in LB and under host mimetic conditions (pooled human urine and RPMI 1640). In general, biofilm formation by phage-resistant UPEC was increased relative to that of parental strains, although not all conditions achieved statistical significance as indicated in figure panels. UTI89-1 (1.9-fold, *P *= 0.049) and UTI89-3 (2.5-fold, *P *= 0.033), demonstrated significantly increased biofilm formation in LB medium, while UTI89-2 demonstrated enhanced biofilm formation in RPMI 1640 (1.2-fold, *P *= 0.032) (Fig. 4E). DS566-1 displayed increased biofilm formation in both LB and RPMI 1640 compared with its wild-type strain (3.6-fold, *P *= 0.036, and 4.7-fold, *P *= 0.021, respectively), whereas DS566-2 had increased biofilm formation in pooled human urine (2.3-fold, *P* = 0.0065) (Fig. 4F).

### Phage resistance is associated with increased antibiotic susceptibility in urine.

**FIG 5:**
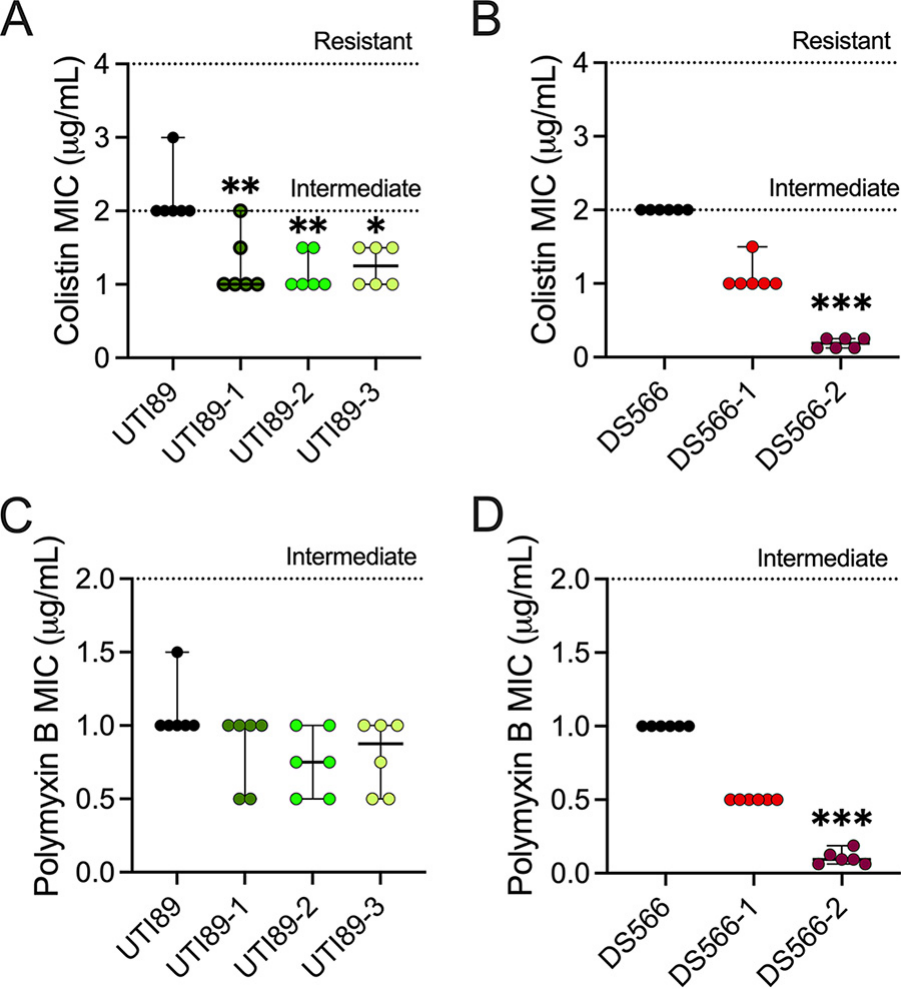
Phage resistance renders UPEC more susceptible to antibiotics that target the bacterial outer membrane. Colistin MICs of UTI89 (A) and DS566 (B) and respective phage-resistant strains in pooled human urine. Polymyxin B MICs of UTI89 (C) and DS566 (D) and respective phage-resistant mutants in pooled human urine. Assays were performed in technical duplicate in six independent experiments, with points on graphs representing mean values of each independent experiment. Bars represent median and 95% confidence intervals. Data were analyzed by Kruskal-Wallis test using Dunn’s multiple-comparison test. *, *P < *0.05; **, *P < *0.01; ***, *P < *0.001.

Since LPS truncation may allow antimicrobials to access the bacterial surface more easily, we compared the susceptibility of wild-type and phage-resistant strains to antibiotics colistin (polymyxin E) and polymyxin B, which both interact with the bacterial outer membrane, in pooled human urine. We observed an approximately 2-fold decrease in the colistin MIC for strains UTI89-1, UTI89-2, and UTI89-3 (Fig. 5A) (*P* = 0.0096, *P* = 0.0040, and *P *= 0.014, respectively) and a nonsignificant change in MIC versus polymyxin B for these same strains (Fig. 5B). In DS566, we observed a 2-fold reduction in MIC for DS566-1 to both colistin and polymyxin B, while DS566-2 had colistin and polymyxin B MICs that were 8- to 16-fold lower than the wild-type strain (0.19 μg/mL and 0.09 μg/mL, respectively) (*P* = 0.0001 and *P* = 0.0001) (Fig. 5C and D). In LB medium, differences between phage-resistant derivatives and parental strains were largely absent (see Fig. S5 in the supplemental material).

10.1128/msphere.00345-22.5 FIG S5 MICs for antibiotics targeting the bacterial outer membrane are not altered when phage-resistant bacteria are grown in LB media. Colistin MICs of UTI89 (A) and DS566 (B) and isogenic phage-resistant mutants. Polymyxin B MICs of UTI89 (C) and DS566 (D) and isogenic phage-resistant mutants. Individual points are representative of independent experiments performed in duplicate. Bars represent median and 95% confidence intervals. Download FIG S5, TIF file, 0.3 MB. Copyright © 2022 Zulk et al. 2022 Zulk et al. https://creativecommons.org/licenses/by/4.0/ This content is distributed under the terms of the Creative Commons Attribution 4.0 International license.

### Phage-resistant UPEC growth is attenuated in urine.

**FIG 6:**
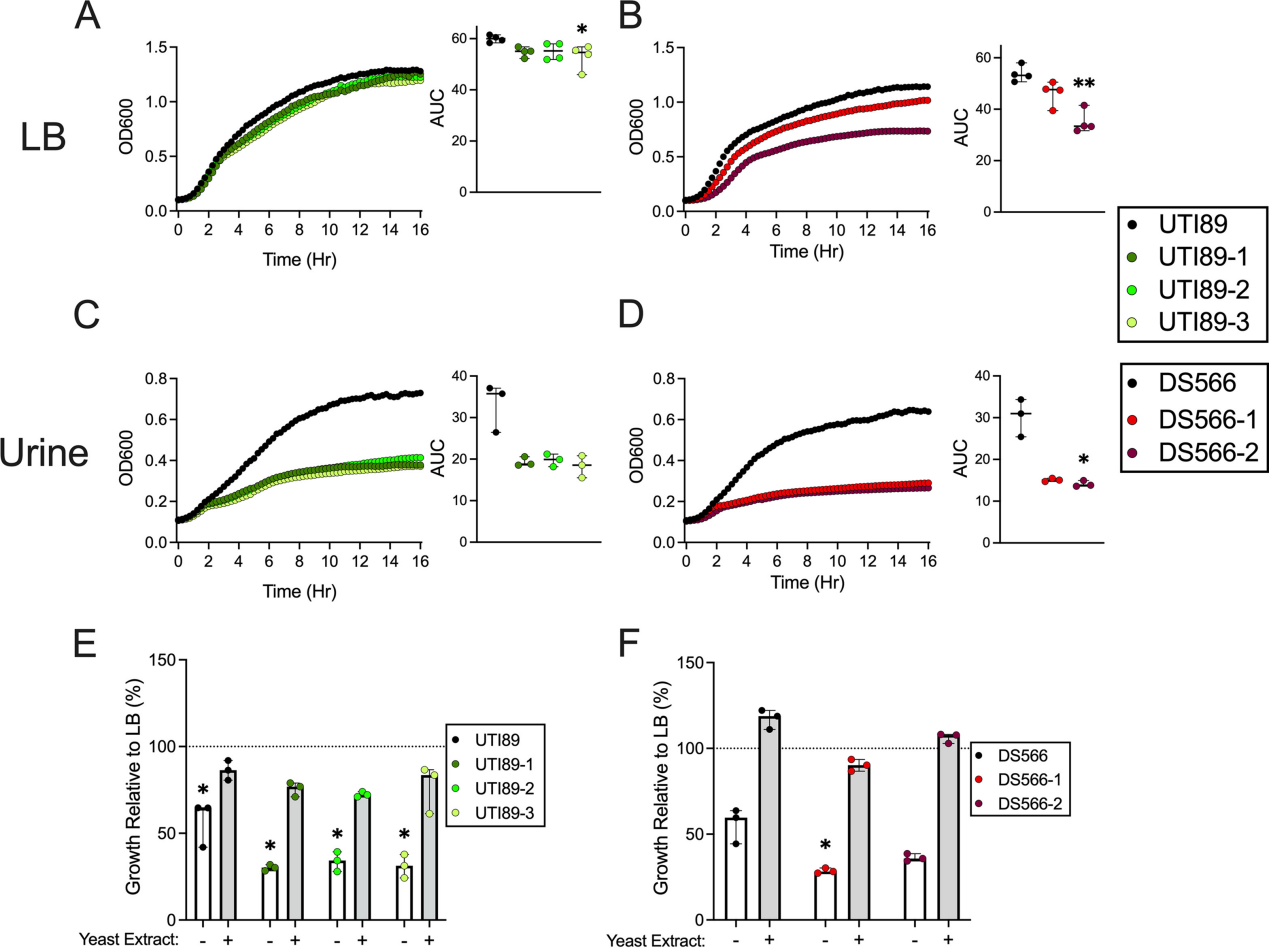
Phage-resistant UPEC growth is attenuated in urine but can be partially restored with nutrient supplementation. WT and phage-resistant strains were grown for 16 h with optical density (left panels) and area under curve (AUC; right panels) displayed. Phage-resistant mutants of UTI89 (A) and DS566 (B) grow similar to parental strains in LB media, but growth is attenuated in pooled human urine (C and D, respectively). UTI89 (E) and DS566 (F) and respective phage-resistant mutants were grown in pooled human urine with or without yeast extract supplementation or LB media for 16 h. Percent growth relative to LB for urine (white bar) and yeast extract-supplemented urine (gray bar) are displayed. Assays were performed in at least technical triplicate in 3 to 4 independent experiments, with points on graphs representing mean values of each independent experiment. Bars represent median with 95% CIs. All data were analyzed by Kruskal-Wallis test using Dunn’s multiple-comparison test. Growth in urine and in urine supplemented with yeast extract was compared with that of the LB control for each strain. *, *P < *0.05; **, *P < *0.01.

To evaluate the impact of phage resistance and associated LPS modifications on bacterial growth, bacteria were grown in either LB medium or pooled human urine for 16 h. While the growth of most phage-resistant bacteria was similar to that of wild-type strains in LB medium (Fig. 6A and B), bacterial growth was severely attenuated in urine across strains regardless of the specific mutations (Fig. 6C and D). Defective growth in urine suggests the possibility that phage-resistant UPEC strains either (i) are more susceptible to active inhibitory factors or (ii) are less fit to grow under nutrient-poor conditions than parental strains. To delineate between these possibilities, we supplemented urine with yeast extract, a primary nutrient source in LB medium. Yeast extract supplementation rescued phage-resistant strain growth to levels resembling growth in LB (Fig. 6E and F). These results suggest that consequences of phage resistance (e.g., LPS deficiency) may directly or indirectly limit nutrient acquisition under nutrient-deplete conditions, such as human urine; however, deciphering this mechanism further is beyond the scope of this study.

### Phage-resistant UPEC strains exhibit decreased bladder colonization.

**FIG 7:**
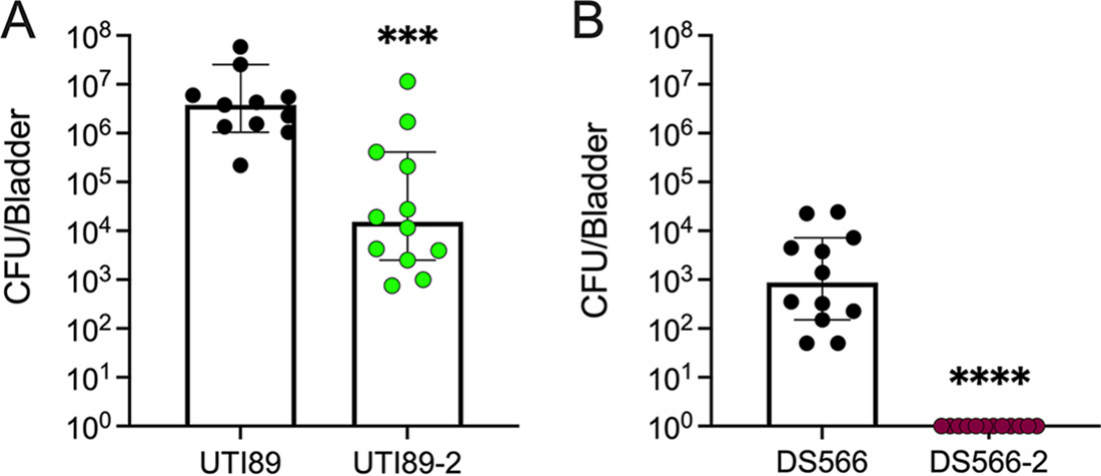
Phage-resistant UPEC strains display decreased bladder colonization in a murine model of UTI. Female C57BL/6J mice were infected transurethrally with 10^8^ CFU of UTI89 or UTI89-2 (A) or DS566 or DS566-2 (B). After 24 h, bladders were removed, and bladder bacterial burdens were assessed. Points represent individual mice (biological replicates) assayed over two separate experiments. (A) UTI89, *n *= 11; UTI89-2, *n *= 12. (B) DS566, *n *= 12; DS566-2, *n *= 12. Lines represent median and 95% confidence intervals. Data were analyzed by Mann-Whitney test. ***, *P < *0.001; ****, *P < *0.0001.

Because phage-resistant strains had comparable or increased biofilm formation, adherence, and internalization to their parental strains *in vitro*, we evaluated the ability of two phage-resistant strains (UTI89-2 and DS566-2) to colonize the mouse urinary tract compared with their parental strains. These strains were selected because they harbor distinct LPS biosynthesis pathway mutations with different degrees of LPS truncation limited to the outer core (UTI89-2) or outer and inner cores (DS566-2). Mice received transurethral bacteria (10^8^ CFU), and at 24 h postinfection, bladder UPEC burdens were quantified. UTI89-2 was significantly attenuated in its colonization compared with wild-type UTI89 (249-fold decrease, *P *= 0.0007) (Fig. 7A). Although wild-type DS566 achieved lower bacterial burdens in the bladder than wild-type UTI89, DS566-2 displayed a potent colonization defect, with bacterial burdens below the limit of detection in all bladders at 24 h postinfection (*P < *0.0001) (Fig. 7B).

## DISCUSSION

Although well-characterized in bacteriologic media, phage-bacterial dynamics are not well-defined in the urinary environment. Here, we identified that (i) in both LB and human urine, resistance arises to two genetically distinct phages with mutations converging on LPS biosynthesis; (ii) phage resistance attenuates UPEC growth in urine, but defective growth is partially rescued by nutrient supplementation; (iii) phage-resistant bacteria are sensitized to membrane-interacting antibiotics in human urine; and finally, (iv) while phage resistance may enhance adherence, invasion, and biofilm formation *in vitro*, it does not translate to heightened bladder colonization *in vivo*. In total, these findings suggest that phage-resistant bacteria may arise during phage therapy for UTI but that the resulting bacteria may be less capable of causing disease and/or sensitized to other treatment options.

Phage resistance is a well-documented phenomenon; however, few studies have assessed phage resistance outside bacteriologic media and in environments relevant to human medicine. Our results demonstrate that UPEC-phage dynamics are similar across environmental conditions but may be more highly influenced by bacterial host genetics as revealed by differences in phage killing kinetics among UPEC strains. In both LB and urine, phage resistance developed within 16 h, and mutations observed in response to HP3 and ES17 phages shared roles in LPS synthesis. In agreement with our study, ExPEC strains have been shown previously to evade HP3 killing by mutating LPS synthesis (35). Additionally, 16C1, an independently derived LPS mutant in UTI89 (54), was also resistant to HP3 and ES17. LPS is a major component of the Gram-negative outer membrane and provides stability and anchoring for other outer membrane proteins (55). LPS mutation in E. coli attenuates several cellular processes, including cellular membrane integrity, resistance to antibiotics, growth in low pH and high detergent conditions, and importantly, resistance to bacteriophage (35, 52, 56–60). Here, we demonstrate that these same detrimental effects occur in the urinary environment among phage-resistant strains. Some of our strains harbor a mutation in transcription factor rfaH, which is involved in the assembly of the LPS (61) but also regulates capsule and alpha-hemolysin among other factors (62). The contributions of these other pathways to phage resistance and associated phenotypes were not examined in the current study.

Urine composition displays dynamic nutrient and solute concentrations throughout the day and between individuals (63–65). Phage-resistant UPEC strains were equally capable of growth in LB as parental strains but grew poorly in urine. This result echoes recent work by García et al. who demonstrated that several genes involved in LPS biosynthesis are important for bacterial growth in urine (66). The addition of yeast extract to urine rescued bacterial growth to levels resembling growth in LB, suggesting that this phenotype may be due ineffective nutrient acquisition or utilization under deplete conditions. In human urine, amino acid and carbohydrate metabolic pathways are upregulated in UPEC compared with iron-limited LB, and mutations in these central metabolic pathways attenuate UPEC fitness *in vivo* (67). Outer membrane proteins, used to transport nutrients through the outer membrane, often form complexes with LPS for assembly and insertion into the membrane (52, 55, 68). Decreased efficacy or number of outer membrane proteins in phage-resistant strains may explain how nutrient supplementation overcomes growth defects in urine; however, the exact mechanisms underlying these results are outside the scope of this study.

Increased biofilm formation occurs in E. coli harboring LPS “deep-rough” mutations *in vitro* (69). Since enhanced biofilms could lead to worse outcomes in patients treated with phages, we assessed if the phage-resistant UPEC displayed this same increased biofilm formation. Partially aligning with previous literature, we observed *in vitro* biofilm increases in UTI89 strains containing *rfaH* mutations, although increased biofilm formation was not observed uniformly across all conditions. Increased biofilm formation was reported in E. coli containing *rfaH* mutations, although differing E. coli strains, biofilm generation, and enumeration methods were used (70). One strain, UTI89-1, also possessed a frameshift mutation in *tsx*, encoding an outer membrane protein and receptor to a T6-like phage (71); however, given the phenotypic similarities between UTI89-1 and the other two phage-resistant UTI89 strains not harboring *tsx* mutations, we hypothesize *tsx* is unlikely to significantly contribute to the observed *in vitro* phenotypes under our experimental conditions. Additionally, we observed increased biofilm formation in phage-resistant DS566 strains that was most pronounced in DS566-2, the most severely attenuated LPS structure based on genomic predictions. Although *in vitro* biofilm assays may not fully reflect the capacity to form biofilms *in vivo*, our results corroborate observations by Nakao et al. who demonstrated enhanced biofilm production by E. coli defective in LPS heptose biosynthesis (69). A limitation of our study is that all phage-resistant strains identified were isolated *in vitro* and yet previous work by our lab suggests that resistance is likely to develop *in vivo* as well (35).

UPEC interactions with the bladder epithelium are crucial for UTI pathogenesis. We observed a minimal impact of phage resistance to HTB-9 cell adherence in the UTI89 background, although adherence was significantly increased in DS566 phage-resistant strains. These results echo work done by Nagy et al. who found no role for *rfaH* in adherence to intestinal cells (72). In contrast, two of three UTI89 phage-resistant strains showed an increased invasion of HTB-9 cells. Similarly, avian pathogenic E. coli strains lacking *rfaH* are more readily engulfed by chicken macrophages than the wild type (73). The mechanism for this increased uptake was not further investigated but could explain the increased invasion of HTB-9 cells.

Since our results suggested that phage resistance could alter host-UPEC interactions, we used a mouse model of UTI to investigate *in vivo* infection outcomes. Phage-resistant strains in both UTI89 and DS566 backgrounds were worse at colonizing the murine bladder than their wild-type counterparts. Similarly, targeted inactivation of *rfaH* dramatically lowers the recovery of UPEC strain 536 from the urinary tract at 21 days postinfection (62). Additionally, Aguiniga et al. used targeted gene deletions to identify LPS domains essential for colonization of the bladder using UPEC strain NU14 (74). While the LPS mutations assessed by this group are not identical to those observed in our study, the functional consequences (e.g., outer membrane truncation and inner membrane truncation) are likely similar. In addition, García et al. have shown recently that *rfaG*, another gene involved in LPS biosynthesis, is important for colonization of the murine bladder (66). Of note, we did not investigate all phage-resistant strains isolated from this study *in vivo*, and it remains possible that alternative phenotypes may be present in other phage-resistant UPEC strains. Although our study does not investigate the immune response to phage-resistant UPEC with LPS modifications, others have observed the “rough” LPS phenotype in several asymptomatic bacteriuria strains, suggesting that these pathogens may be less virulent in humans (75). In fact, asymptomatic bacteriuria isolates with truncated LPS, among other virulence gene mutations, have been suggested as possible competitors which could be intentionally introduced in patients to prevent recurrent UPEC infection (76–79). In agreement with our study, bladder colonization using these UPEC strains is not always achieved despite frequent bacterial inoculations, suggesting a possible colonization defect of these strains (79).

Phage and antibiotics have the potential to work together synergistically (43, 80). Indeed, we observed that phage-resistant UPEC strains were more sensitive to antibiotics targeting the bacterial membrane in urine, a condition that was not investigated previously. Colistin and polymyxin B both permeabilize the bacterial outer membrane by interacting with lipid A (81). As observed in the biofilm and adherence and invasion assays, enhanced susceptibility to antibiotics was most pronounced in DS566-2, the strain with the most significant predicted LPS truncation. Although Gram-negative pathogens lacking the lipid A portion of LPS are resistant to colistin (82, 83), phage-driven mutations in our strains are predicted to retain lipid A.

In summary, bacteria quickly become resistant to phage under urinary tract conditions; however, mutations providing phage resistance may come at the cost of bacterial fitness and ultimately reduce pathogenesis in the urinary environment. Many other targets for phage exist in addition to LPS (38) raising the appealing possibility that combining phages with distinct targets could both reduce bacterial burden and attenuate bacterial virulence of emerging antibiotic-resistant bacteria. These findings expand our knowledge of phage resistance of UPEC in the context of the urinary tract and support its continued development as a target for controlling UTI.

## MATERIALS AND METHODS

### Bacterial strains and mammalian cell lines.

Uropathogenic E. coli strains UTI89 (84), CFT073 (ATCC 700928) (85), DS515, and DS566 (accession CP092534) were used to isolate bacteriophage-resistant bacteria. DS515 and DS566 are urinary isolates from spinal cord injury patients with neurogenic bladders at the Michael E. DeBakey VA Medical Center (Houston, TX). UTI89 strain 16C1 harbors a transposon insertion within the *rfa* operon and has been described previously (54). All bacterial strains were grown overnight, shaking, at 37°C prior to experiments. Phages HP3 (accession KY608967) and ES17 (accession MN508615) were isolated from environmental sources and wastewater, respectively (24, 31). A human bladder epithelium carcinoma cell line (ATCC HTB-9) was grown in RPMI 1640 (Corning) containing 10% heat-inactivated fetal bovine serum (FBS) at 37°C with 5% CO_2_ and was passaged every 3 to 5 days.

### Bacteriophage preparation.

Purified HP3 and ES17 stocks were prepared as described previously (47), their titers were determined, and they were stored in phage buffer (35) at 4°C until use.

### Human urine pool preparation.

Urine samples were collected from six healthy male and female volunteers, 20 to 50 years old, under approval of the Baylor College of Medicine (BCM) institutional review board (IRB) (protocol H-47537). Following collection, urine was warmed to 37°C and filtered (0.22 μm) before storage at 4°C.

### UPEC growth in the presence of phage.

Overnight UPEC cultures were diluted 1:100 in LB or pooled human urine and added to 96-well microtiter plates. For phage challenge, bacteriophage preparations diluted in phosphate-buffered saline (PBS) were added at an MOI of 0.1, 0.001, or 0.00001 to reach a final volume of 150 μL. Control bacterial wells were treated with PBS alone. Growth at 37°C was measured by OD_600_ every 15 min for 18 h under aerobic shaking conditions using a Tecan Infinite 200 plate reader. Relative bacterial growth was determined by calculating the percent OD_600_ of a given well compared with the mean of bacterium-only wells at that same time point.

### Isolation of phage-resistant bacteria.

Overnight bacterial cultures were diluted 1:100 in fresh LB or pooled human urine and challenged with phage (MOI, 0.1) in a 150-μL total volume. OD_600_ was measured every 15 min as described above. Buffer (PBS) without phage was a control for noninfected bacterial growth. After 18 h, phage-treated wells with bacterial growth were streaked onto soft agar overlay plates containing phage. This process was repeated once for colonies which grew on phage top agar to isolate clonal populations. Phage-resistant isolates were confirmed by phage spot assay onto lawns of UPEC.

### Purification and visualization of LPS.

UPEC LPS was isolated through hot aqueous-phenol extraction following methods described previously (86). Extracted LPS samples (15 μL) were run at 120 V for 1.5 h on 4% to 12% SDS-polyacrylamide gels and stained using the Pro-Q Emerald 300 lipopolysaccharide gel stain kit (Molecular Probes) following the manufacturer’s directions. Gels were visualized on a ProteinSimple AlphaImager HP system.

### Generating bacterial growth curves for phage-resistant bacteria in LB and urine.

For growth assessments of phage-resistant bacteria, overnight cultures were diluted 1:100 in LB media or pooled human urine and added to 96-well microtiter plates (100 μL). Growth at 37°C was measured by OD_600_ every 15 min for 16 h under aerobic shaking conditions using a BioTek Cytation 5 plate reader (Gen5 v3.10).

### Biofilm assays.

Bacterial biofilms were quantified as described previously with minor adaptations (87). Overnight cultures were diluted to an OD_600_ of 0.1 in LB, urine, or RPMI 1640 before 200 μL of it was added to 96-well tissue culture plates. Plates were incubated under stationary, aerobic conditions at 37°C for 24 h, and the OD_600_ was measured to quantify overall bacterial growth. Nonadherent bacteria were removed, and biofilms were washed three times with PBS and dried at 55°C for 1 h. Crystal violet (0.2%, 200 μL) was added, and plates were incubated at room temperature for 30 min. Crystal violet was removed, and biofilms were washed five times with PBS. To release the crystal violet, 200 μL of an 80:20 mixture of ethanol and acetone was added to each well. The released crystal violet solution (100 μL) was transferred to a new 96-well plate and absorbance measured at OD_595_ on a BioTek Cytation 5 instrument.

### Adherence and invasion assays.

Adherence and invasion assays were performed as described previously (88, 89). Briefly, HTB-9 cell confluent monolayers were washed, and 400 μL of fresh RPMI 1640 medium was added. Mid-log-phase bacterial cultures (OD_600_, 0.4) were diluted 1:10 in PBS, and 100 μL of the bacterial dilution was added to wells. To facilitate bacterial-cell contact, plates were spun at 200 × *g* for 2 min and incubated at 37°C in 5% CO_2_. For adherence assays, after 30 min, cells were washed six times with PBS before the addition of 100 μL of 0.025% Trypsin-EDTA. Cells were incubated at 37°C for 7 min, after which time, 400 μL of 0.025% Triton X-100 was added to each well to lyse HTB-9 cells. The contents of the wells were pipetted up and down 25 times before being diluted and plated onto LB agar. For invasion assays, infected HTB-9 cells were incubated with bacteria for 2 h before the medium was removed and replaced with 500 μL of RPMI 1640 medium containing 50 μg/mL gentamicin. The cells were again incubated for 2 h before being washed and lysed as described for adherence assays.

### MIC assays.

MIC assays were conducted as described previously (90). Briefly, overnight cultures of bacteria were subcultured to mid-log phase (OD_600_, 0.4 to 0.6) before being pelleted and resuspended 1:10 in PBS. Antibiotics were diluted in LB or urine and added to 96-well plates. Bacteria were added at a 1:10 dilution to each well before being incubated under stationary, aerobic conditions at 37°C overnight. To measure metabolic activity, resazurin (Sigma-Aldrich) was added at a concentration of 6.75 μg/mL to each well and plates were incubated for an additional 3 h at 37°C. Fluorescence, indicated by a resazurin-to-resofurin conversion, was measured using an excitation/emission of 550 nm/600 nm on a BioTek Cytation 5 instrument. MIC was determined as the lowest antibiotic concentration at which a >90% reduction in fluorescent signal was observed compared with no-antibiotic controls. MIC breakpoints were obtained from the Clinical and Laboratory Standards Institute (CLSI) (91).

### Murine UTI model experiments.

All animal experiments were approved by the Baylor College of Medicine (BCM) Institutional Animal Care and Use Committee (protocol AN-8233) and were performed under accepted veterinary standards. Female C57BL/6J mice (strain number 000664) were purchased from Jackson Laboratories or from BCM vivarium stock, and all experiments were conducted when mice were aged 8 to 12 weeks. Animals were allowed to eat and drink *ad libitum* throughout the duration of experiments. An established murine UTI model was used as described previously (89, 90). Mice were anesthetized with inhaled isoflurane, and approximately 10^8^ CFU of bacteria suspended in PBS was instilled transurethrally into the bladders of mice in a 50-μL volume. At 24 h postinfection, bladders were removed and homogenized in tubes containing 1.0-mm-diameter zirconia/silica beads (Biospec Products; catalog number 11079110z) using a MagNA Lyser instrument (Roche Diagnostics). Serial dilutions of homogenized organs were plated on LB agar and enumerated the following day.

### Sequencing and analysis of phage-resistant UPEC.

DNA was isolated from overnight bacterial cultures using the E.Z.N.A. bacterial DNA kit (Omega Bio-Tek) following the manufacturer’s instructions. Sequencing was performed by Novogene using the Illumina platform. Reads were trimmed to Q30 and a minimum length of 50 bp using BBDuk (v38.84). Phage-resistant isolates were compared with wild-type strains using three methods, as follows: (i) reads were independently assembled *de novo* using Geneious assembler (Geneious 2022.0.1). Contigs of resistant bacteria were then compared with parental contigs using progresssiveMauve (version 26 February 2015) and disagreements extracted (92); (ii) Reads from phage-resistant bacteria were mapped to wild-type bacteria from method 1, followed by variant analysis using Geneious 2022.0.1 variant finder. (iii) Resistant bacteria were compared with parental bacteria using snippy-multi script in Snippy (93). Snippy results agreeing with methods 1 and 2 are presented. For reference selection in Snippy, bacterial reads were validated through the EDGE bioinformatic (v2.4.0) software phylogenetic analysis module, using RAxML and a prebuilt E. coli single nucleotide polymorphism (SNP) database (94, 95). The published UTI89 genome (accession CP000243.1) was used as a reference. Since DS566 did not cluster with another strain closely enough to be used as a reference, it was further categorized using Center for Genomic Epidemiology MLST 2.0 software (v2.0.4; database version 18 October 2021) which predicted it belonged to sequence type 1193 (ST1193) (96, 97). E. coli MCJCHV-1 (accession CP030111.1) was chosen as a reference for DS566. Variants present in both parental strains and resistant progenies were discarded. Circular diagrams were made using CGView Server and modified using Microsoft PowerPoint (98).

### Statistics.

*In vitro* experiments were performed at least three times independently with at least one technical duplicate. Mean values of independent experiments were used to represent biological replicates for statistical analyses. *In vivo* experiments were conducted at least twice independently with individual mice serving as biological replicates. Experimental data were combined prior to statistical analyses. Mann-Whitney tests were used to compare murine bladder colonization (Fig. 7A and B). The Kruskal-Wallis test with Dunn’s multiple comparisons was used to compare adherence and invasion differences between the strains (Fig. 4A to D), to assess changes in MIC values (Fig. 5A to D), and to analyze bacterial growth in LB and urine (Fig. 6A to F). Two-way repeated-measures analysis of variance (ANOVA) with Geisser-Greenhouse correction and Dunnett’s multiple comparisons was used for biofilm experiments (Fig. 4E and F) and for analysis of bacterial growth in the presence of phage (Fig. 3C and D). Statistical analyses were performed using Prism, v9.2.0 (GraphPad Software Inc., La Jolla, CA). *P* values of <0.05 were considered statistically significant.

### Data availability.

Assembled wild-type DS566 (accession CP092534) and phage-resistant UTI89-1 (accession CP092531), UTI89-2 (accession CP092530), UTI89-3 (accession CP092529), DS566-1 (accession CP092533), and DS566-2 (accession CP092532) sequencing data generated from this study are available in NCBI GenBank under project number PRJNA808067.
