# Supplementary figures and images for: Phage Resistance Accompanies Reduced Fitness of Uropathogenic Escherichia coli in the Urinary Environment
Source: mSphere. 2022 Aug 3;7(4):e00345-22. doi: 10.1128/msphere.00345-22 (PMC9429881; doi:10.1128/msphere.00345-22)

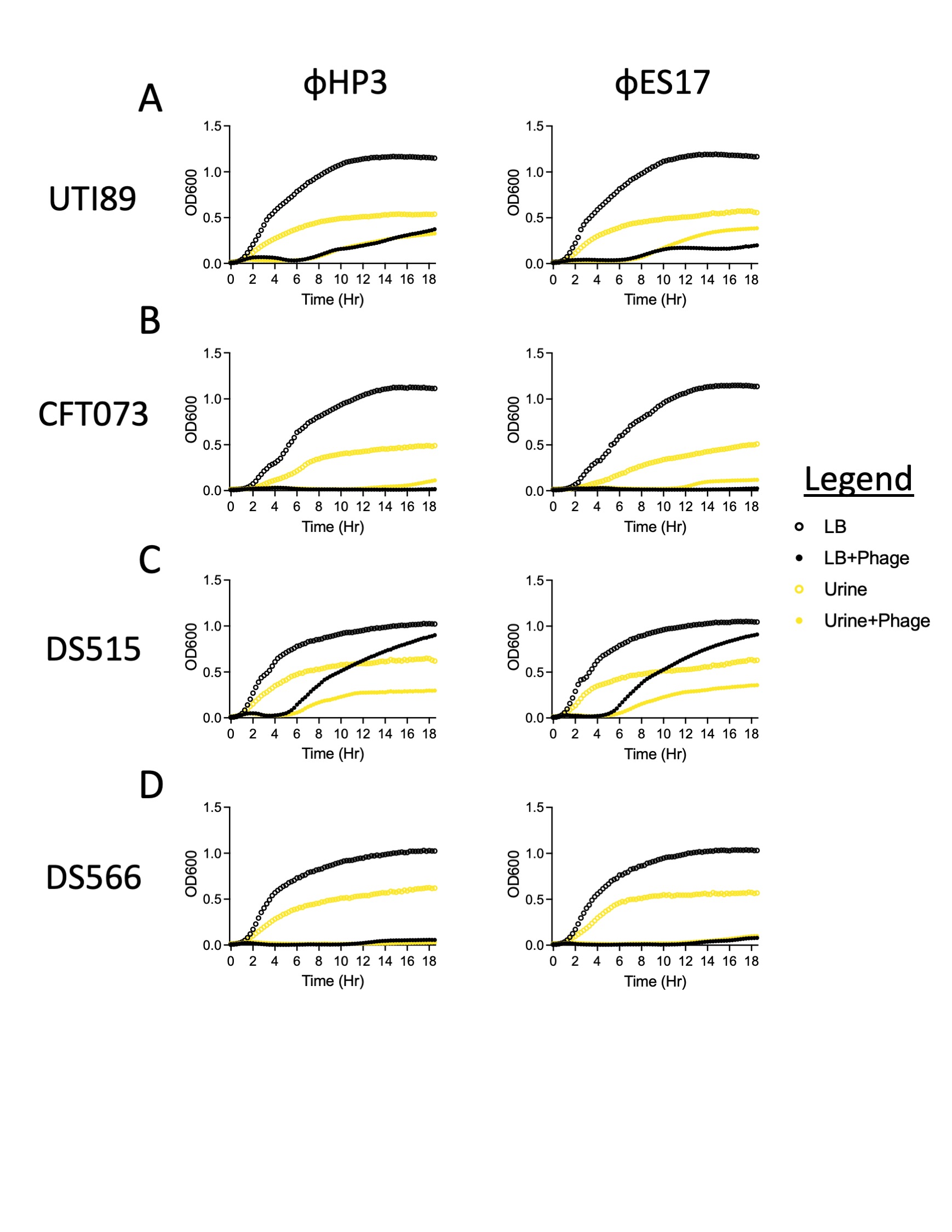

Supplement: FIG S1 [file msphere.00345-22-s0002.jpg]

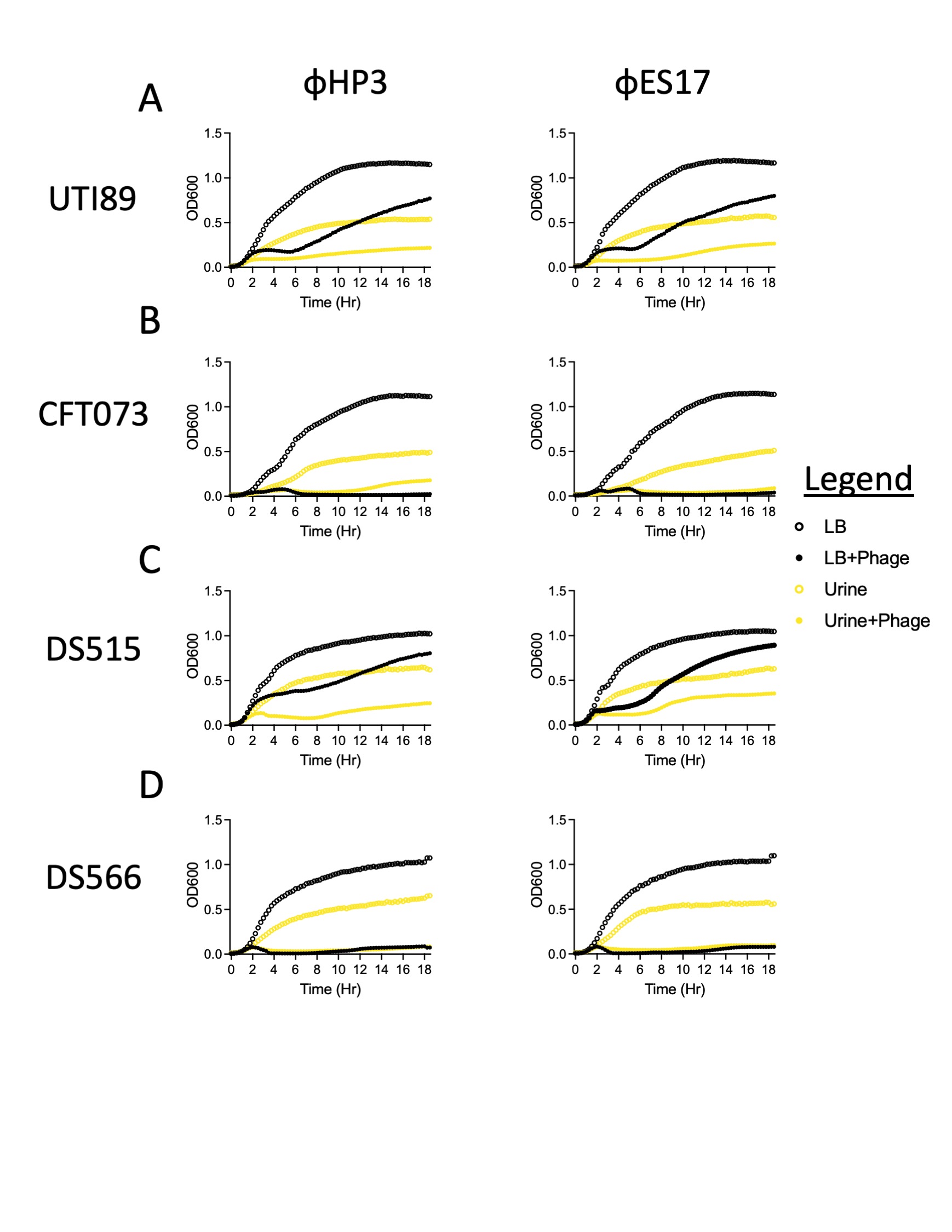

Supplement: FIG S2 [file msphere.00345-22-s0003.jpg]

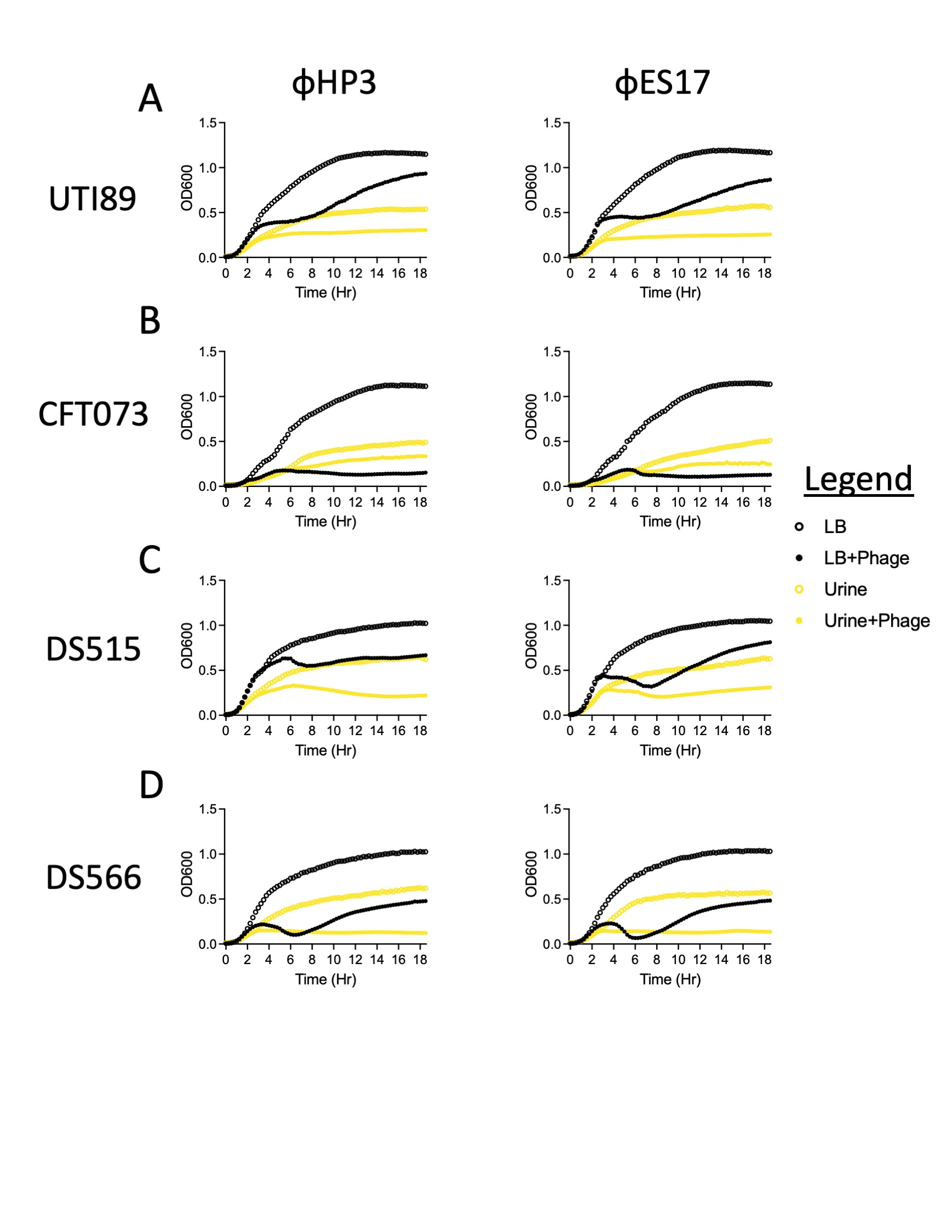

Supplement: FIG S3 [file msphere.00345-22-s0004.jpg]

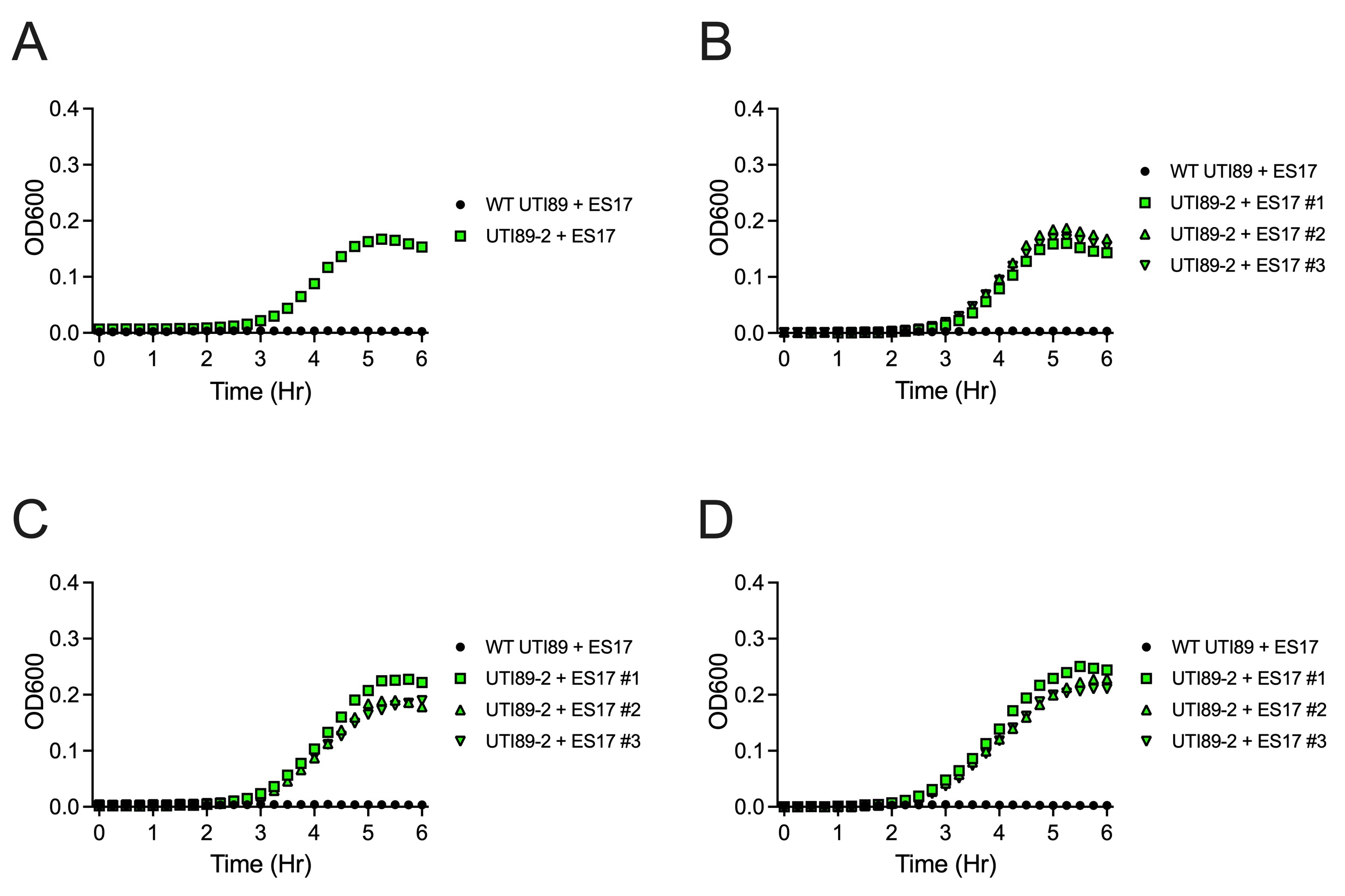

Supplement: FIG S4 [file msphere.00345-22-s0005.jpg]

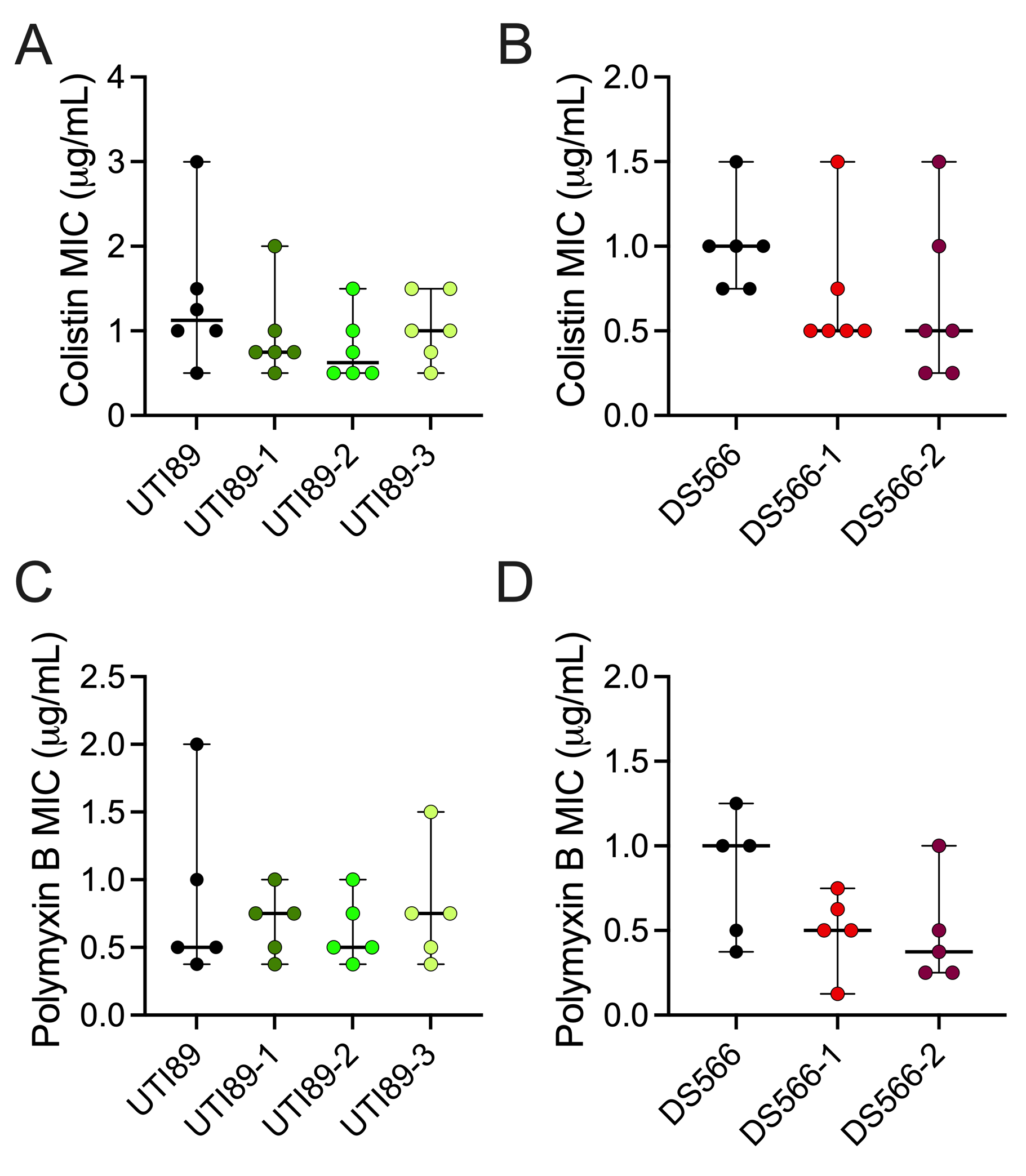

Supplement: FIG S5 [file msphere.00345-22-s0006.tif]
