# Supplementary material for: Phage Resistance Accompanies Reduced Fitness of Uropathogenic Escherichia coli in the Urinary Environment
Source: mSphere. 2022 Aug 3;7(4):e00345-22. doi: 10.1128/msphere.00345-22 (PMC9429881; doi:10.1128/msphere.00345-22)
Supplement: TABLE S1 [file msphere.00345-22-s0001.docx]

**Supplemental Tables:**

**Supplementary Table 1: Non-LPS associated mutations identified during sequencing of phage-resistant isolates.** Site and type of non-LPS biosynthesis-associated mutations observed in phage-resistant strains arising from *in vitro* screening and identified by whole genome sequencing.
